# Critical Limb Ischemia in a Young Man: Saddle Embolism or Unusual Presentation of Thromboangiitis Obliterans?

**DOI:** 10.1155/2013/830540

**Published:** 2013-12-03

**Authors:** Federico Bucci, Adriano Redler, Leslie Fiengo

**Affiliations:** ^1^Vascular Surgery Department, Sud Gironde Community Hospital, rue Langevin, 33210 Langon, France; ^2^General and Vascular Surgery Department, “Umberto I” University Hospital, Viale del Policlinico, 00186 Rome, Italy

## Abstract

Thromboangiitis obliterans (TAO), also known as Buerger's disease, is a rare cause of peripheral arterial disease in western countries. Tobacco smoking is strongly correlated to the pathogenesis of this inflammatory vascular disease. We report the case of a 32-year-old tobacco and cannabis consumer presenting with right critical limb ischemia. Computerized tomography angiography revealed a bilateral tibioperoneal arterial occlusion and an aortoiliac saddle embolus. The patient was treated with intravenous heparin, transcatheter thrombolysis, and selective Fogarty embolectomy. Instrumental and laboratory examinations revealed that patient's most likely diagnosis was TAO. Arterial embolism is uncommon in Buerger's disease but should be always excluded in these patients.

## 1. Introduction

Thromboangiitis obliterans (TAO), also known as Buerger's disease (BD), is a rare cause of peripheral arterial disease (PAD) in western countries. Reportedly, annual incidence of TAO is 12.6 per 100,000 representing only 0.5% of all causes of PAD. Tobacco is essential in promoting and maintaining this disease and 95% of patients affected by TAO are smokers [1].

## 2. Case Report

**Figure 1:**
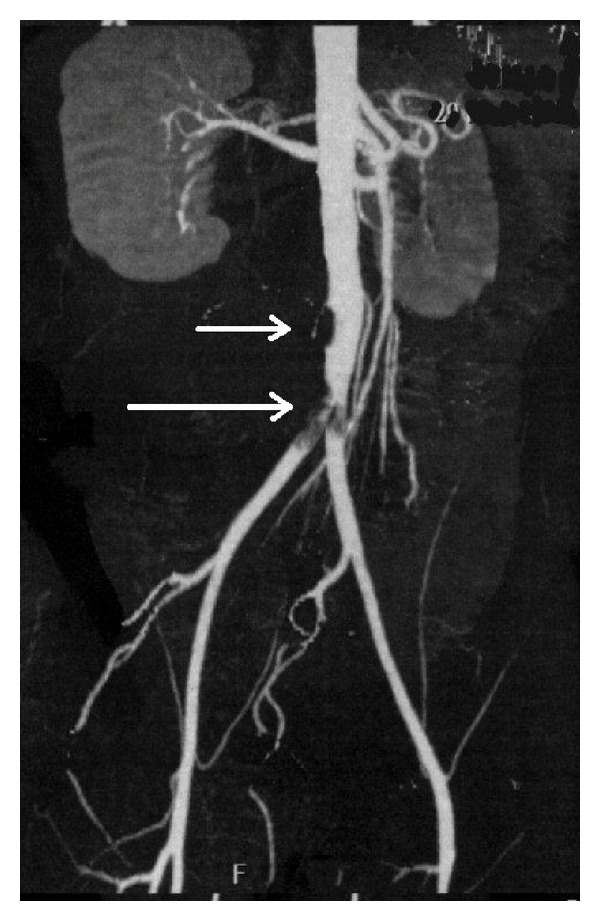
CT angiography showing the presence of an intraluminal aortic (short arrow) and iliac (long arrow) saddle embolus.

**Figure 2:**
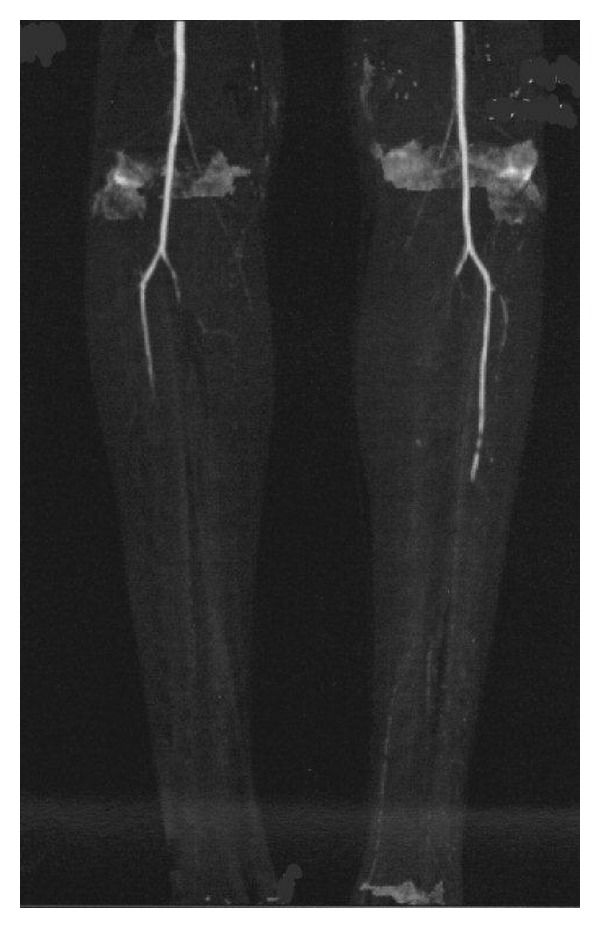
CT angiography of the lower limbs showing distal posterior and anterior tibial artery occlusion of the left side and three-vessel occlusion of the right side.

**Figure 3:**
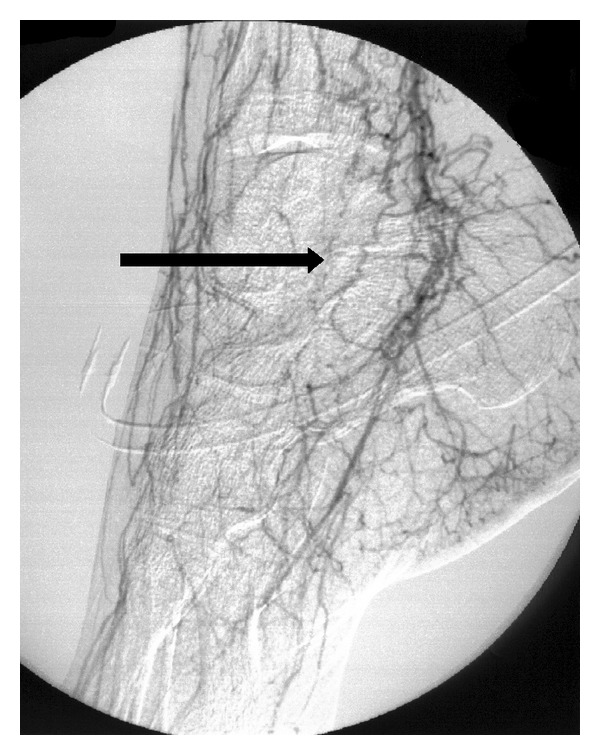
Intraoperative arteriography showing the distal occlusion of right tibial and peroneal arteries, with the typical “corkscrew” collateral arteries (black arrow), suggestive of a vasculitis.

A 32-year-old man was referred to the emergencies of our hospital because of a right lower limb critical limb ischemia. Past medical history included chronic alcoholism and a three-month history of bilateral intermittent claudication. He did not report any episode of superficial thrombophlebitis. He smoked about 10 cigarettes since the age of ten and 10 cannabis joints daily since the age of twelve. He had no other cardiovascular risk factors. At clinical examination, his right leg was extremely painful and pale. He had absent pedal pulses on both sides, and a mild sensory loss on the right side. Allen's test of upper extremities was negative. Echo Doppler was suggestive of a bilateral common iliac occlusion and of a three-vessel occlusion on the right leg. A computerized tomography (CT) angiography detected the presence of an intraluminal aortic and iliac clot (Figure 1) and a bilateral distal tibial vessels occlusion (Figure 2). The patient was then fully anticoagulated with intravenous heparin. A transthoracic echocardiogram was also performed and did not detect any proximal source of emboli. The patient was then operated on: under general anesthesia, a right iliofemoral embolectomy associated to a selective right popliteal, tibial, and peroneal embolectomy and intraoperative intraarterial thrombolysis of tibial vessels. During the operation, no thrombus was found in the infrapopliteal vessels, but intraoperative arteriography showed a diffuse narrowing associated to total occlusion at the ankle with the typical “corkscrew” collateral arteries suggestive of a chronic vasculitis (Figure 3). BD was then suspected. The postoperative period was uneventful, with complete remission of symptoms. The aortoiliac embolus was sent to bacteriology and some *Micrococci* were found. Subsequently, the patient was treated with medical therapy including full dose low molecular weight heparin, antiplatelets, and pentoxifylline, and a smoking-cessation program was started. A control thoracic and abdominal angio-CT scan, done also in order to detect a proximal source of embolism, showed the absence of residual aortoiliac clot, but the chronic occlusion of the anterior tibial and peroneal arteries bilaterally. The contralateral lower limb did not require any operation. After discharge the patient underwent laboratory tests looking for diabetes and thrombophilia that were unremarkable. These included factor II and V mutation, disorders of plasminogen activation, ATIII deficiency, protein C and protein S deficiency, and homocysteine serum levels. Extensive autoimmune testing looking for autoimmune disorders potentially responsible for thrombotic events including anti-lupus erythematosus, antinuclear, antimitochondrial, and anti-phospholipids antibodies were all negative. We then concluded that the patient was affected by BD.

Anticoagulation was stopped. On the last visit at 12 months, the patient has recently restarted smoking about five cannabis joints every day; he still presents a right-sided intermittent claudication with long walking distances. Control angio-CT scan was unchanged if compared to the last one realized at hospital.

## 3. Discussion

TAO is a nonatherosclerotic inflammatory occlusive disease that affects small and medium-sized arteries and veins of upper and lower extremities. The role of tobacco as the most important etiopathogenic factor of TAO is well established, probably because of an idiosyncratic autoimmune response to some of its components [1]. Some authors suggest that addictions such as cannabis and cocaine may be coresponsible for Buerger's disease, accelerating its clinical presentation and aggravating its extension [2, 3]. Genetic predisponding factors are probably relevant as well. In fact, this vasculitis is more frequent in East Europe, Middle East, and Asia, with the highest prevalence documented in the Ashkenazi Jews population [4, 5]. Recurrent periodontal infections could play a role [6]. In our patient's case bacteriological tests of the removed thrombus revealed the presence of some bacteria belonging to the former genus *Micrococcus*, but we cannot exclude that it was a contamination of the thrombus during the operation.

TAO usually concerns young men, but its prevalence is increasing in women because patterns in smoking are changing, with an increasing number of female smokers. The most common age of presentation is during the fourth decade. TAO usually affects the distal infrapopliteal arteries, but iliac and femoral localization of the disease have also been described. Clinical presentation of TAO usually starts with coldness, pallor, and paraesthesia of the extremities. Intermittent claudication, whenever present, usually lasts for a short period. Critical limb ischemia occurs at a more advanced stage of the disease and is the most common clinical presentation of TAO on admission. Episodes of superficial migratory thrombophlebitis may also be referred [1, 3]. Thromboembolism is uncommon in TAO and differential diagnostics with diabetes, hypercoagulable states, and autoimmune abnormalities predisposing to thrombosis such as lupus erythematosus, antiphospholipid syndrome, and sclerodermia are strongly recommended [7]. The presence of a proximal or a cardiac source of embolism should always be excluded as well.

The occlusion of infrapopliteal vessels is a potentially limb-threatening event that can be particularly dramatic in this population as TAO usually presents on young patients. Long-term outcome is very poor with an amputation risk of 38% at ten years if smoking is not discontinued [8]. Because of this, the use of psychological support should be considered.

Diagnostic workup can include Doppler US, computerized tomography, magnetic resonance imaging, and standard angiography. In our opinion, standard arteriography is still an excellent tool, especially in cases where there is diagnostic doubt.

Most of uncomplicated TAO patients are currently managed with antiplatelets and vasodilators, such as calcium-channel blockers and alpha-blockers. In a recent small study, Bosentan, an orally administered molecule normally used to treat pulmonary arterial hypertension in systemic sclerosis patients, showed good short and mid-term results to treat TAO [9]. Another option is represented by prostanoids such as Iloprost, a strong vasodilator that is administered intravenously on an inpatient basis. Iloprost seems to be safe and effective to relief rest pain and increase wound healing in case of critical limb ischemia [10]. Percutaneous interventions could theoretically play a role for TAO patients in case of desperate limb salvage, but long-term results are still unclear [11]. The role of bypass surgery is very controversial and rarely possible, with poor results at medium and long-term because of the absence of a viable distal vascular bed [12]. Intra-arterial thrombolytic agents such as urokinase or recombinant tissue plasminogen activator (rtPA) may occasionally be used in the acute phase of the disease and whenever, as in our patient's case, there is evidence of fresh thrombus.

Other typologies of treatment such as peripheral sympathectomy, hyperbaric oxygen therapy, and spinal cord stimulation seems to be effective especially to treat rest pain, but to this day have not clearly proved their efficacy [5, 13]. Therapeutic neoangiogenesis with autologous bone marrow mononuclear cell implantation showed promising short- and mid-term results [14, 15], but its value remains to be demonstrated in the long period.

Regarding our reported case, the presence of an aortoiliac fresh thrombus and of a consequent distal embolization made the diagnosis quite challenging. According to Olin's criteria [1] elements suggestive of TAO were first of all patient's history of a heavy smoker since extremely young age. Secondly, the angiographic finding of a total occlusion of tibial and peroneal vessels at the ankle with abundant collaterals, described as “corkscrew,” suggestive of a chronic occlusive process (Figure 3). The suspicion of BD was basically confirmed by the differential diagnostic work up: a transthoracic echocardiogram and a thoracoabdominal CT angiography were performed and did not detect any proximal source of emboli. Once clot's removed a postoperative angio-CT confirmed that the abdominal aorta, the bifurcation and the iliacs were totally normal. All the laboratory blood tests (diabetes mellitus, hypercoagulability states, autoimmune diseases, and connective tissue disorders) were unremarkable as well. In the authors' opinion the most likely is the coexistence of two separate entities, the vasculitis, and the thromboembolic phenomena, in the same patient, rather than a manifestation of BD. Even if theoretically smoking may exacerbate the disease by increasing platelet aggregation and clot's formation [16], the cause of this saddle embolus remains unclarified.

## References

[1] Olin JW (2000). Thromboangiitis obliterans (Buerger’s disease). *The New England Journal of Medicine*.

[2] Grotenhermen F (2010). Cannabis-associated arteritis. *The Journal of Vascular Diseases*.

[3] Martin-Blondel G, Koskas F, Cacoub P, Sne D (2011). Is thromboangiitis obliterans presentation influenced by cannabis addiction?. *Annals of Vascular Surgery*.

[4] Kobayashi M, Nishikimi N, Komori K (2006). Current pathological and clinical aspects of Buerger’s disease in Japan. *Annals of Vascular Surgery*.

[5] Joviliano EE, Dellalibera-Joviliano R, Dalio M, Évora PR, Piccinato CE (2009). Etiopathogenesis, clinical diagnosis and treatment of thromboangiitis obliterans—current practices. *International Journal of Angiology*.

[6] Iwai T, Inoue Y, Umeda M (2005). Oral bacteria in the occluded arteries of patients with Buerger disease. *Journal of Vascular Surgery*.

[7] Lee T, Seo JW, Sumpio BE, Kim SJ (2003). Immunobiologic analysis of arterial tissue in Buerger’s disease. *European Journal of Vascular and Endovascular Surgery*.

[8] Cooper LT, Tse TS, Mikhail MA, McBane RD, Stanson AW, Ballman KV (2004). Long-term survival and amputation risk in thromboangiitis obliterans (Buerger’s disease). *Journal of the American College of Cardiology*.

[9] de Haro J, Acin F, Bleda S, Varela C, Esparza L (2012). Treatment of thromboangiitis obliterans (Buerger’s disease) with bosentan. *BMC Cardiovascular Disorders*.

[10] Fiessinger JN, Schäfer M (1990). Trial of iloprost versus aspirin treatment for critical limb ischaemia of thromboangiitis obliterans. *The Lancet*.

[11] Graziani L, Morelli L, Parini F (2012). Clinical outcome after extended endovascular recanalization in Buerger’s disease in 20 consecutive cases. *Annals of Vascular Surgery*.

[12] Sasajima T, Kubo Y, Inaba M, Goh K, Azuma N (1997). Role of infrainguinal bypass in Buerger’s disease: an eighteen-year experience. *European Journal of Vascular and Endovascular Surgery*.

[13] Piazza G, Creager MA (2010). Thromboangiitis obliterans. *Circulation*.

[14] Miyamoto K, Nishigami K, Nagaya N (2006). Unblinded pilot study of autologous transplantation of bone marrow mononuclear cells in patients with thromboangiitis obliterans. *Circulation*.

[15] Franz RW, Shah KJ, Johnson JD (2011). Short- to mid-term results using autologous bone-marrow mononuclear cell implantation therapy as a limb salvage procedure in patients with severe peripheral arterial disease. *Vascular and Endovascular Surgery*.

[16] Hung J, Lam JYT, Lacoste L, Letchacovski G (1995). Cigarette smoking acutely increases platelet thrombus formation in patients with coronary artery disease taking aspirin. *Circulation*.

